# Comparison of caries lesion detection methods in epidemiological surveys: CAST, ICDAS and DMF

**DOI:** 10.1186/s12903-018-0583-6

**Published:** 2018-07-06

**Authors:** Ana Luiza Sarno Castro, Maria Isabel Pereira Vianna, Carlos Maurício Cardeal Mendes

**Affiliations:** 1 Department of Health, State University of Feira de Santana, Transnordestina, s/n, Novo Horizonte, Feira de Santana, Bahia CEP 44036-900 Brazil; 2 Department of Public Oral Health, School of Dentistry, Federal University of Bahia, Araújo Pinho, 62, Canela, Salvador, Bahia CEP 40110040 Brazil; 3 Postgraduate Studies in Interactive Processes of Organs and Systems, Health Science Institute, Federal University of Bahia, Avenida Reitor Miguel Calmon, 1272, Salvador, Bahia CEP 40231300 Brazil

**Keywords:** Epidemiology, DMF index, ICDAS, CAST, Epidemiological surveys

## Abstract

**Background:**

Although dental caries is a globally widespread disease, there is no consensus regarding the method that should be used for their detection. In recent decades, a variety of new methods have been proposed for measuring caries in a population. Three caries detection methods, the decayed, missing and filled (DMF) index, the International Caries Detection and Assessment System (ICDAS) and the Caries Assessment Spectrum and Treatment (CAST), were compared to provide information to guide future method choices.

**Methods:**

This was a descriptive, cross-sectional study in which three methods were used to measure caries in students, staff and their dependents at UNEB (State University of Bahia), Salvador, Brazil. We compared the mean application time of each method and the frequencies obtained by each method using the following indicators: the most severe caries lesion per individual; the mean number of missing, filled and decayed teeth; and the disease extent.

**Results:**

The mean time taken to apply the DMF was 3.8 min; for ICDAS, it took 8.9 min, and for CAST, 4.7 min. When calculating the indicator the most severe caries lesion per individual, the prevalence rates were as follows: 28.1% for DMF, 84.0% for ICDAS and 75.0% for CAST. The mean numbers of decayed, missing and filled teeth were 6.0 according to the DMF, 6.2 according to ICDAS and 5.9 according to CAST. When the disease extension indicator was used, the following percentages of teeth were affected by caries: DMF 22.12%, ICDAS 49.11% and CAST 33.2%.

**Conclusions:**

The DMF underestimated the occurrence of caries lesions in individuals but was the fastest method to apply. ICDAS obtained detailed information regarding lesion severity, but it was a time-consuming method and difficult to analyse. CAST described disease distributions very well and identified lesion severities and preventive and curative needs in the examined group, and the time required to apply CAST was similar to that of the DMF.

**Electronic supplementary material:**

The online version of this article (10.1186/s12903-018-0583-6) contains supplementary material, which is available to authorized users.

## Background

Caries is a disease that is present in all countries around the world. The signs and symptoms accumulate with increasing age, with a prevalence of 100% in most adult populations [1]. The occurrence of caries is a major cause of pain, tooth loss, aesthetic and functional problems and work absenteeism [2].

Although caries is an oral disease that occurs worldwide, according to Baelum and Fejerskov [3], there is no consensus regarding which criteria and methods should be used for its detection, and few studies compare different methods for measuring caries in epidemiological surveys involving adult populations [4, 5].

In recent decades, a wide variety of new methods have been developed to measure caries in a population [6–13]. These methods measure caries lesions based on different diagnostic thresholds. Some methods are able to measure early non-cavitated enamel lesions, which are observed only after drying the tooth surface, as indicated in the International Caries Detection and Assessment System (ICDAS) [11]. Others can detect early non-cavitated enamel lesions without the need to dry the tooth surface, as indicated in the Caries Assessment Spectrum and Treatment (CAST) [14]. The method used most frequently since the 1940s is the decayed, missing and filled (DMF) index [15], which has generally been used to detect caries from dentin lesions. This change in diagnostic threshold affects the results of the prevalence rates calculated using each method.

The limitations of the DMF index have been noted in the literature, and therefore, new indexes were developed, among them the ICDAS and CAST have stood out, which were validated and used in several countries and therefore were chosen for comparison with the DMF [1, 8, 16–22].

This study aimed to compare the ICDAS, CAST and the DMF in terms of their operational aspects and their capacity to determine the extent and severity of dental caries in the same sample of individuals, thus informing future choices in regard to which caries detection method should be used in a community. To the best of our knowledge, this is the first study to compare the three methods at the same time in an adult population.

## Methods

### Study design and sample type

This was a descriptive cross-sectional study, in which three caries measurement methods were applied in the same group. The students, employees and their dependents, who were attending the Medical, Dental and Social Service (SMOS) at the State University of Bahia – UNEB, located in Salvador, Bahia, Brazil, during the period from September 6 to December 13, 2016, were examined. Because it was a convenience sample, no inferential statistics were calculated [23]. Only adults are provided care by this dental service, and the inclusion criterion was being in the care of the service during the aforementioned period; therefore, all examined individuals were adults, and 73% were between 18 and 31 years of age.

### Ethical aspects

The individuals were examined after being properly informed about the procedures of the study and signing informed consent forms. The work was approved by the Research Ethics Committee of the Sciences Institute of the Federal University of Bahia under CAAE number 48500115.2.0000.5662.

### Examiner calibration

Four examiners, with the aid of three note-takers, applied the three caries detection methods to the study population. Calibration of the four examiners was performed from August 8 to August 26, 2016. Each week, professors were asked to teach about a particular method and guide the examiner calibration. Eighteen hours of training were devoted to each method, and a total of 54 h of examiner calibration training was provided.

On the morning of the first day of training, there was a lecture on the CAST method, followed in the afternoon by training with projected photos and with in vitro teeth, in ceramic pots with modelling clay. On the morning of the next day, five patients were examined by the four study examiners, who were supervised by an experienced examiner, the method criteria were discussed, and the hits and errors of the four examiners were compared until a consensus was reached on the classification of the conditions diagnosed in the patients. In the afternoon, five more patients were examined to calculate the inter-examiner Kendall’s Coefficient of Concordance (Kendall’s W) [24]. These same patients were examined three days later to calculate the intra-examiner Kendall’s Coefficient of Concordance. The following week, the same procedure was performed for ICDAS, adding the use of two e-learning programmes, one from the site ICDAS.org [25] and the other from a training programme developed by Port and Zaleski [26]. In the third week, the same procedure used in the calibration of CAST was followed for the DMF index training.

CAST was used according to the recommendations of the method’s manual [27]. ICDAS was used as recommended on the ICDAS.org site at the time of the examination [25]. The DMF was applied according to the manual of the latest epidemiological survey of the Brazilian Ministry of Health [28], with the diagnostic threshold for the decayed tooth component in the DMF measured in non-cavitated dentine lesions (codes 4, 5 and 6 of ICDAS and 4, 5 and 6 of CAST). Descriptions of all the codes for the three methods are provided in the Additional file 1: Appendix.

### Data collection procedure

The examinations were conducted in the period from September 6, 2016, to December 13, 2016. The selected exam period corresponded to the period of greatest frequency in the classroom by the university students in the second semester of the academic year. An exam schedule was drawn such that each examiner used a different method each week, the first to examine prophylaxis with brush and floss to remove plaque and allow a better clinical examination for him and the other examiners. Three dental offices were used. The patients, when they arrived, were provided with explanations about the research, signed the informed consent, and received prophylaxis from the first examiner. The fist examiner applied one method, the patient remained in the chair and another examiner examined him. The examiners then alternated, each one applying a different method. Dental equipment (dental light, dental chair) and dental instruments (WHO probe and plain dental mirror) were used. A compressed air syringe was used to dry the teeth during application of the ICDAS, which is the only method that requires the use of this resource.

During the examinations, each examiner applied a different method each week, and 780 forms were completed using the three methods (each of the 260 patients was examined three times in the same session by different examiners applying ICDAS, CAST and the DMF so that each patient produced three forms). Re-examinations were performed in 10% of the sample for each method to compare the intra-examiner and inter-examiner reproducibility using Kendall’s W Coefficient of Concordance [24].

### Analysis method

The proportions and absolute, percentage and standardised mean differences obtained with each method were compared. The data were entered into Microsoft Excel (2007) and analysed in R [29].

The tooth surface was the analysis unit used to calculate Kendall’s W (i.e., Kendall’s coefficient of concordance). For each individual, 128 surfaces (those of 28 permanent teeth) were examined. The third molars were not included in the examination. Surfaces with sealants were regarded as healthy.

Kendall’s W Coefficient of Concordance is suitable for testing the reliability of ordinal data and thus was used to test the intra- and inter-examiner agreement. This coefficient has the advantage of not being affected by the prevalence of the studied object [24].

The most severe caries lesion per individual was used as an indicator to identify those in need of restorative and preventive treatment. This indicator shows the highest caries score in the carious component observed in an individual; therefore, it shows the greatest need for treatment at the moment of examination.

For example, an individual with restorations, extracted teeth and enamel caries will be included as an individual who needs preventive treatment to prevent this enamel lesion from developing into a dentine lesion, and another individual with enamel caries and a lesion that reached the pulp will be classified as an individual with caries that can produce an odontogenic abscess. Thus, a priority for the service would be to attend to this individual. This manner of classifying the individual’s situation and not the mean number of teeth affected by caries facilitates communication between planning epidemiologists and other professionals in the health area. The most severe caries lesion per individual was determined based on the maximum score per subject used by the group of researchers who developed CAST [30].

This indicator was used in the same way that prevalence calculations were performed by CAST [27]; that is, per individual, without considering the calculation of sealants, fillings and teeth extracted due to caries. A modification was incorporated relative to CAST, which was the inclusion of enamel caries lesions when calculating disease prevalence.

Initially, data were recorded for the tooth surface. The most severe caries lesion per tooth was then recorded, according to the worst conditions encountered when using each method, and finally, the worst lesion observed per individual was recorded.

When calculating the most severe caries lesion per individual, scores related to sealants, fillings, extractions and excluded surfaces were ignored. The order of severity adopted, from lowest to highest, was as follows: non-cavitated enamel lesions detected after drying the tooth; non-cavitated enamel lesions detected without drying the tooth; cavitated enamel lesions; non-cavitated dentine lesions; cavitated dentine lesions; filled surface with cavitated dentine lesions and extensive dentine lesions with pulp involvement.

Differences in the percentages of individuals classified as having caries lesions according to each method calculated with the indicator of the most severe caries lesion were compared.

The methods were also compared in terms of extension of disease, which, according to Maltz et al. [31], is the number of teeth or surfaces affected by the disease.

Three units of measure were used according to the different purposes: the surface unit to be further detailed and sensitive was used to calculate the concordance between the examinees, the tooth unit was used to compare the extent that the caries reached the group, and the individual unit used to differentiate people according to the worst condition found by calculations using the indicator the most severe caries lesion per individual.

When using the tooth as the measurement unit, lesions found by each method were classified as pre-morbidity (enamel lesions), morbidity (non-cavitated dentine and cavitated dentine lesions), severe morbidity (dentine lesions that had reached the pulp) and mortality (extracted teeth) according to the CAST Manual classification [27].

The means for decayed, missing and filled teeth obtained by each method were compared. The data obtained from CAST and ICDAS can be converted into the mean DMF if the same diagnostic threshold applied for the caries lesion is used for all three methods to classify the tooth.(In the study, the diagnostic threshold was established for all three methods from D3, i.e., non-cavitated dentine lesions and cavitated dentin lesions) [25]. Thus, we can add the following: teeth classified as carious; teeth identified as filled; and teeth lost due to caries. The total can then be divided by the number of examined persons to calculate a mean of decayed, filled and lost teeth using each method.

To facilitate comparisons between the methods and to simplify reporting of the results obtained by ICDAS, all the method codes relating to fillings were grouped together such that the codes distinguishing various types of fillings were reported simply as filled.

In the present study, ICDAS was used as recommended on the ICDAS.org site at the time the study was conducted, without incorporating Exposed Pulp, Ulceration, Fistula, Abscess (PUFA), International Caries Classification & Management System (ICCMS) or Lesion Activity (LA) measures.

To calculate the application time of each index, each examination was timed from the first annotated code to the last recorded code. The mean times spent performing examinations using each of the different methods were calculated and compared using the mean percentage difference, which is the difference between two values divided by the average of the two values shown as a percentage.

The standardised mean difference (SMD) was also utilised, which expresses the size of the intervention effect in each study relative to the variability observed in that study. To interpret the SMD data, Cohen’s d was used. Cohen’s d is a rather simple statistical expression: specifically, it reflects the difference between two group outcomes divided by the population standard deviation and is represented in the following formula: d = (μ1 ‐ μ2)/*σ* [32, 33].

$$ \mathrm{SDM}=\frac{\mathrm{Difference}\ \mathrm{in}\ \mathrm{mean}\ \mathrm{outcome}\ \mathrm{between}\ \mathrm{groups}}{\mathrm{Standard}\ \mathrm{deviation}\ \mathrm{of}\ \mathrm{outcome}\ \mathrm{among}\ \mathrm{participants}} $$

## Results

### Overall intra- and inter-examiner agreement and general sample characteristics

Most of the examined individuals were students (70.3%), female (74.2%) and aged 18 to 31 years (73.0%), with a mean age of 28 and a standard deviation (SD) of 10 years.

The Kendall’s W values found in the intra- and inter-examiner tests demonstrated a very good level of agreement, as Kendall’s W values above 0.90 were observed for the four examiners during application of the methods [34]. All values of Kendall’s W for the different examiners are provided in the Additional file 1: Appendix.

### Comparison of methods in regard to the application time

The coefficient of variation (CV) is a standardised measure of dispersion expressed as a percentage; it is the ratio of the standard deviation to the mean, shows the extent of variability in relation to the mean, and is used to compare the heterogeneity of the number of applications found with different methods. As shown in Table 1, the fastest method was the DMF, in which the mean application time was 3.8 min. However, the DMF also presented the most heterogeneous results (39.5% coefficient of variation). The results for CAST were similar to those of the DMF, with a mean application time of approximately one additional minute (4.7 min). This method presented the least heterogenous data (coefficient of variation 29.8%). ICDAS took longer than both the DMF and CAST, with a mean of 8.9 min. Its coefficient of variation was similar to that of CAST (31.5%).

**Table 1:** Mean application times, in minutes, of the ICDAS, CAST and DMF methods

Method	Mean (SD)	CV (%)	Min–Max
CAST	4.7 (1.4)	29.8	1.5–7.0
DMF	3.8 (1.5)	39.5	1.2–8.2
ICDAS	8.9 (2.8)	31.5	3.3–18.0

*CV*  coefficient of variation, *SD*  standard deviation, *Min*  minimum, *Max*  maximum

Table 2 reveals that the biggest differences observed were between the DMF and ICDAS (134.2% and 2.3) methods, while the smallest differences were found between the CAST and the DMF methods (23.7% and 0.6).

**Table 2:** Differences in the mean application times of the ICDAS, CAST and DMF methods

METHODS	Absolute mean difference (minutes)	Percentage mean difference (%)	Standardised mean difference
CAST X DMF	0.9	23.7	0.6
CAST X ICDAS	4.2	47.2	1.9
DMF X ICDAS	5.1	134.2	2.3

To interpret the magnitude of the standardised mean difference, Cohen [32] recommended thresholds based on standardised mean differences: 0.2 reflects a small effect; 0.5 reflects a medium effect; and greater than 0.8 reflects a large effect. Using these guidelines, the results indicate that the effect sizes show large differences between the ICDAS and both the DMF and CAST and a medium difference between CAST and the DMF.

### Comparison between methods according to the most severe caries lesion per individual

The distribution of caries prevalence in the population was determined according to the indicator of the most severe caries lesion per individual. This prevalence includes individuals who had caries lesions according to the diagnostic threshold of each method. When this indicator was calculated using the DMF index, a caries prevalence of 28.1% was found among the individuals. However, when using ICDAS, a prevalence of 84.6% was found, and when CAST was applied, the prevalence was 75.0% (the prevalence corresponds to the complement of the absence of lesions shown in the first row of Table 3).

**Table 3:**
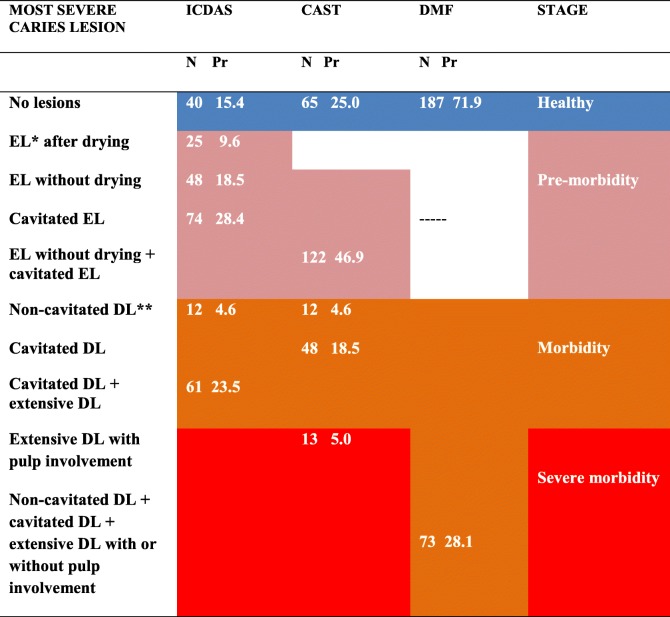
Distribution of indicators of the most severe caries lesions per individual according to the ICDAS, CAST and DMF methods

*EL  Enamel lesion ** *DL*  Dentine lesion, *Pr*  Prevalence

According to the CAST and ICDAS methods, the worst condition observed in approximately half of the individuals was enamel caries lesions (46.9 and 56.5%, respectively). These individuals were classified as having no caries lesions when using the DMF (Table 3).

Table 3 shows that when using the DMF method, 28.1% of the individuals had at least one dentine lesion, although the severity of each lesion was not indicated. When ICDAS and CAST were used, 4.6% of the individuals showed teeth with non-cavitated dentine damage as their worst condition (these individuals are in the morbidity group).

According to the ICDAS, 23.5% of the patients had extensive or cavitated dentine lesions. CAST differentiated these lesions into two groups: the morbidity group, comprising the 18.5% of individuals who had at least one tooth with a cavitated dentine lesion and the severe morbidity group, comprising the 5% of individuals who had at least one tooth with an extensive dentine lesion with pulp involvement, which requires more complex treatment.

### Comparison between methods according to the disease extent

In addition to prevalence, these methods can also reveal the extent of caries in a population. According to the results, 77.88% of the examined teeth were classified as healthy when using the DMF, 66.80% when using CAST and 59.11% when applying ICDAS (Table 4).

**Table 4:**
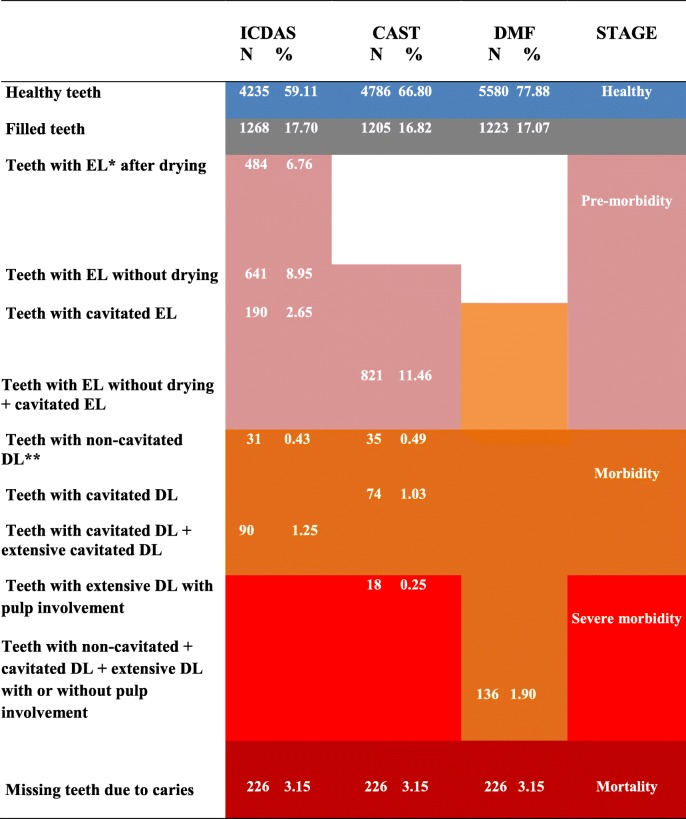
Distributions of classifications of tooth conditions according to the ICDAS, CAST and DMF methods

* *EL*  Enamel lesion ** *DL*  Dentine lesion

According to the examinations performed using ICDAS, 484 teeth (6.76%) showed the first clinical signs of caries, that is, an enamel lesion after drying the tooth; 641 (8.95%) had enamel lesions that could be observed without drying the teeth; and 190 teeth (2.65%) had cavitated enamel lesions. The last two conditions are grouped in CAST as enamel lesions, and 821 teeth were classified as having this condition (11.46%). Teeth with such lesions may be classified as being in the pre-morbidity stage.

Application of the DMF classified 136 teeth as having dentine caries lesions (1.90%), with no distinction between teeth with morbidity or severe morbidity and with all being classified as decayed teeth.

According to ICDAS, 121 teeth were found to be in the morbidity stage: 31 with non-cavitated dentine lesions (0.43%) and 90 with extensive or non-cavitated dentine lesions (1.25%). When using CAST, 109 teeth were diagnosed as being in the morbidity stage; 35 teeth with non-cavitated dentine lesions (0.49%), 74 teeth with cavitated dentine lesions (1.03%) and 18 teeth (0.43%) in the severe morbidity stage with extensive dentine lesions with pulp involvement.

According to the three methods, 226 teeth extracted due to caries (3.15%) were classified as being in the mortality stage. A total of 1268 (17.70%) teeth were filled when examined by ICDAS; 1205 (16.82%) when using CAST and 1223 (17.07%) according to the DMF. The different percentages were due to differences in inter-examiner classifications.

Only 19 teeth (9 people) had sealants, justifying the decision to classify teeth in this condition as healthy. There were no teeth with abscesses or fistulae, and as such, CAST code 7 was not used.

### Comparison between methods according to the mean number of decayed, missing and filled teeth

When calculating the mean DMF for the study group, a value of 6.0 was obtained, which was very similar to the mean of 5.9 calculated using the data obtained from CAST and similar to the sum of teeth classified as decayed, missing and filled according to ICDAS, which was 6.2.

## Discussion

The results of this study showed that most of the individuals examined were students (70.39%) and female (74.2%).

The four examiners obtained Kendall’s W values greater than 0.90. According to Silva et al. [34], a Kendall’s W value equal to or greater than 0.90 indicates that the evaluators applied essentially the same standard, so the three methods presented good reproducibility. This result demonstrated that more detailed methods, including enamel lesions and various codes for dentine lesions, do not diminish reproducibility provided that a good calibration of the examiners is performed.

Regarding differences in application times of the indices, according to Cohen [32], the effect sizes show a large difference between the ICDAS and the DMF [35] and a medium difference between the CAST and the DMF; therefore, choosing the ICDAS instead of the CAST to replace the DMF will have a sizable impact on the increased time necessary to carry out the survey.

According to the literature, the DMF is simple to apply, and analysis of its results is straightforward [1, 36], as also observed in this study.

Several authors [8, 16–19] have stated that the CAST method offers a simple application and analysis. No difficulties were encountered when applying the method during calibration and clinical examinations, nor during the analysis. This method proposes 10 codes arranged in a hierarchical manner, without the need to dry the teeth, contributing to its greater ease of application. On average, it took one minute longer than the time taken to apply the DMF index.

Similar to the present work, CAST and DMF showed an equivalent time in the study by Souza et al. [8], in which an absolute and percentage difference of 1.6 and 2.5% were reported between these two methods, respectively; these are small differences despite the ability of CAST to measure lesions in enamel and distinguish the three levels of gravity of the lesions in dentin.

In a study by Braga et al. [22] using the DMF and ICDAS on the same group of 252 children, ICDAS took twice as long to use in deciduous teeth, and the data generated using this method for cavitated dentine caries lesions were comparable to those of the DMF.

According to the literature, the most complex method to use is ICDAS [18, 20, 21] because its two-digit system uses the first digit to indicate fillings and sealants and the second for the detection of caries lesions. Additionally, enamel lesions are classified according to three different levels. During the examinations, it was necessary to classify each surface in relation to these two digits and to observe each surface before and after drying the tooth, as described below. All 128 tooth surfaces of each patient were analysed without drying the surface to detect the presence of an ICDAS code 2 lesion (enamel lesion without drying). Each surface was then dried for 5 s to detect the presence of ICDAS code 1 lesions (enamel lesion after surface drying). These extra steps meant that this method took longer to apply (8.9 min), taking an average of more than 5 min longer than the DMF.

The total duration of the examinations of the 260 individuals was 4 h longer when using CAST than when using the DMF and 22 h longer when examining patients using ICDAS than when using the DMF. Assuming a hypothetical population of 2600 people, which is 10 times larger than the studied sample, application of the ICDAS would require an additional 220 h, demanding more human resources and making this diagnosis costlier and more time-consuming.

The disadvantages of ICDAS have been reported by de Amorim et al. [21] and Iranzo-Cortes et al. [4], as also verified in the present study. Use of a two-digit system and many codes made it difficult to analyse data, and the application of air to dry surfaces made the method time-consuming.

The development and use of the indicator of the most severe caries lesion per individual aimed to identify people who had enamel caries lesions and those with dentine lesions. This indicator does not include the past history of filled and extracted teeth; instead, it is reversible; that is, it can be zero if all curative and preventive needs of the studied community are met.

This indicator aims to identify the needs of each individual at the time of the examination, and if the worst condition was an enamel lesion, that individual would be identified as belonging to the group needing care for this type of lesion. If a dentine injury is present, the individual will be included in the group for which restoration should be performed. The lesion is not reversible, but the need for treatment for this lesion is reversible; therefore, after the group of individuals receive the treatment, the information related to this dental care will be provided through a more direct measure showing the changes occurring in the community after receiving dental care.

This concept of a reversible measure is employed in CAST. Upon calculating the disease prevalence, the authors [27] recommend considering filled teeth, those with sealants and those without signs of caries as healthy. Therefore, the CAST code values decrease as the population receives dental care, unlike the DMF, for which the mean value does not decrease after treatment.

CAST and ICDAS were used at the enamel level, and the DMF was used at the dentine level. Therefore, these two methods were likely to produce higher caries estimates than the DMF, not because of how they are constructed but because of the much larger number of enamel caries than dentine caries. If the DMF was used with the inclusion of enamel caries, then the difference would substantially decrease. The DMF was used in this way because caries in dentine is the diagnostic threshold commonly employed by the DMF. The different criteria used by the methods can cause variations in the prevalence. This study intends to demonstrate the consequences of the use of certain criteria in the diagnosis of caries and dental care planning.

When using the DMF, a good proportion of the individuals considered to be affected by caries was related to past history (fillings and extractions), and as these conditions were not included, it was concluded that the vast majority of individuals had no need for treatment at the time of examination. In contrast to the DMF, the use of CAST allowed the identification of individuals who had caries lesions on enamel. In addition, CAST was the only method that distinguished lesions with morbidity and severe morbidity in which caries lesions reached the tooth pulp.

The greater detailing of dentine lesions obtained when using CAST has also been described by de Souza et al. [8], who stated that a disadvantage of the DMF is its inability to distinguish between dentine lesions that can be restored and those that require more complex treatment. This limitation precludes the acquisition of an overview of the type of treatment required by a population, thus preventing proper planning with respect to the quantity of dental materials, human resources, methods and equipment required to adequately resolve the situation.

When using the tooth as the measurement unit, ICDAS and CAST showed lower percentages of healthy teeth than the DMF because these methods include enamel caries lesions among their criteria, which is why some teeth classified as healthy by the DMF would be classified as having enamel lesions by other methods.

The examined population had access to dental care at UNEB’s dental service, which might explain the large percentages of teeth with fillings. In the 2010 Brazilian dental health survey, the restored component represented 43.5% of the examined teeth that were affected by caries in the adult population [36]. In the present study, the restored component, according to DMF, represented 77.6% of the teeth affected by caries, which is well above the national average. Access to dental care may also explain the small percentages of teeth with dentine caries lesions.

The ICDAS was the only one of the three methods in which caries lesions were detected in enamel after drying the teeth, which was the major difference observed between ICDAS and CAST; however, the identification of these lesions is one of the reasons for the more time-consuming and laborious nature of ICDAS. The detection of this type of lesion is questionable for use in large population groups because many of the surfaces with these lesions return to a healthy state without any type of treatment and entail a high cost due to the use of prophylaxis, good illumination and compressed air.

Comparability is an important advantage of the DMF; the DMF average can be compared with studies from the 1940s onwards and with data collected from across the world. The mean values obtained using the three methods for decayed, missing and filled teeth were very close. Therefore, to stop using CAST and ICDAS due to a lack of comparability is unreasonable, as the results obtained using these methods can be converted into the mean DMF.

## Conclusions

CAST was able to classify the severity of lesions and identify preventive and curative needs without the need to dry the tooth, with a similar application time to the DMF. Therefore, CAST is suitable for use in the detection of caries in a population.

The prevalence calculated by the DMF was lower than that detected by the other methods because this index does not include enamel lesions, and DMF does not distinguish between severities of caries lesions, which is important for health planning. However, the DMF is a quick, simple and easily applied index that can be used when it is not necessary to measure enamel lesions.

Finally, the ICDAS method obtained the most detailed data regarding caries classification, but it is difficult to use in epidemiological surveys of dental caries, as it is time-consuming. Moreover, its analysis is complex because it classifies enamel lesions into three levels and uses two digits and too many codes. However, it was the only method among the three able to detect the first clinical signs of caries and can be used in conjunction with other systems to assess caries activity. Therefore, the ICDAS method is appropriate for use in clinical studies and in individual evaluations of caries lesions.

## Additional file

Additional file 1: Appendices: Methods codes and intra-examiner and inter-examiner reproducibility. (DOCX 25 kb)

## Abbreviations

CAST: Caries Assessment Spectrum and Treatment
DMF: Decayed, Missing and Filled index
ICCMS: International Caries Classification & Management System
ICDAS: International Caries Detection and Assessment System
LA: Lesion Activity
PUFA: Exposed Pulp, Ulceration, Fistula, Abscess
SMOS: Medical, Dental and Social Service
UNEB: State University of Bahia

## References

[1] Fejerskov O, Kidd E (2009). Dental caries: the disease and its clinical management.

[2] Petersen PE, Bourgeois D, Ogawa H, Estupinan-Day S, Ndiaye C (2005). The global burden of oral diseases and risks to oral health. Bull World Health Organ.

[3] Baelum V, Ole F, Fejerskov O, Nyvad B, Kidd E (2015). How big is the problem? Epidemiological features of dental caries. Dental caries: the disease and its clinical management.

[4] Iranzo-Cortés JE, Montiel-company JM, Almerich-Silla JM (2013). Caries diagnosis: agreement between WHO and ICDAS II criteria in epidemiological surveys. Community Dent Health.

[5] Melgar RA, Pereira JT, Luz PB, Hugo FN, Araujo FB (2016). Differential impacts of caries classification in children and adults: a comparison of ICDAS and DMF-T. Braz Dent J.

[6] Mount GJ, Hume WR (1997). A revised classification of carious lesions by site and size. Quintessence Int.

[7] Fisher J, Glick M (2012). FDI world dental federation science committee. A new model for caries classification and management: the FDI world dental federation caries matrix. J Am Dent Assoc.

[8] de Souza AL, Leal SC, Bronkhorst EM, Frencken JE (2014). Assessing caries status according to the CAST instrument and WHO criterion in epidemiological studies. BMC Oral Health.

[9] Nyvad B (2004). Diagnosis versus detection of caries. Caries Res.

[10] Castro ALS, Vianna MIP, Reis SR de A (1999). A new index for measuring dental caries: reversible dental caries index-IRCD. Rev Fac Odontol Univ Fed Bahia.

[11] Ismail AI, Sohn W, Tellez M, Amaya A, Sen A, Hasson H (2007). The international caries detection and assessment system (ICDAS): an integrated system for measuring dental caries. Community Dent Oral Epidemiol.

[12] Sheiham A, Maizels J, Maizels A (1987). New composite indicators of dental health. Community Dent Health.

[13] Monse B, Heinrich-Weltzien R, Benzian H, Holmgren C, Helderman WP (2010). PUFA–an index of clinical consequences of untreated dental caries. Community Dent Oral Epidemiol.

[14] Frencken JE, de Amorim RG, Faber J, Leal SC (2011). The caries assessment Spectrum and treatment (CAST) index: rational and development. Int Dent J.

[15] Klein H, Palmer CE, Knutson JW (1938). Studies on dental caries: I. Dental status and dental needs of elementary school children. Public Health Rep.

[16] Maciel IP. Epidemiological survey of oral health in schoolchildren using the instrument CAST. 2016. http://repositorio.unb.br/bitstream/10482/21254/1/2016_IsadoraPassosMaciel.pdf. Accessed 9 Feb 2017.

[17] Baginska J, Rodakowska E, Milewski R, Kierklo A (2014). Dental caries in primary and permanent molars in 7-8-year-old schoolchildren evaluated with caries assessment Spectrum and treatment (CAST) index. BMC Oral Health..

[18] Baginska J, Rodakowska E, Wilczko M, Kierklo A (2016). Caries assessment Spectrum and treatment (CAST) index in the primary molars of 6- to 7-year-old polish children. Oral Health Prev Dent.

[19] Anchala K, Challa R, Vadaganadham Y, Kamatham R, Deepak V, Nuvvula S (2016). Assessment of dental caries in primary dentition employing caries assessment spectrum and treatment index. J Orofac Sci.

[20] Almerich-Silla JM, Boronat-Ferrer T, Montiel-Company JM, Iranzo-Cortés JE. Caries prevalence in children from Valencia (Spain) using ICDAS II criteria, 2010. 2014. https://www.ncbi.nlm.nih.gov/pmc/articles/PMC4259373/. Accessed 14 Mar 2017. 10.4317/medoral.19890 PMC4259373 25350591

[21] de Amorim RG, Figueiredo MJ, Leal SC, Mulder J, Frencken JE (2012). Caries experience in a child population in a deprived area of Brazil, using ICDAS II. Clin Oral Investig.

[22] Braga MM, Oliveira LB, Bonini GA, Bonecker M, Mendes FM (2009). Feasibility of the international caries detection and assessment system (ICDAS-II) in epidemiological surveys and comparability with standard World Health Organization criteria. Caries Res.

[23] Ludwig DA (2005). Use and misuse of p-values in designed and observational studies: guide for researchers and reviewers. Aviat Space Environ Med.

[24] Kendall MG. Rank correlation methods. 1948[cited on march 17, 2017]; Available at: https://onlinelibrary.wiley.com/doi/abs/10.1111/j.2044-8317.1956.tb00172.x

[25] Topping GVA, Hally JD, Bonner BC, Pitts NB (2008). Training for the international caries detection and assessment system (ICDAS II): CD-room and web-based educational software.

[26] Port AL da F, Zaleski V Development of a digital learning object for caries lesions detection training using ICDAS [cited on March 1, 2017]; Available at: https://www.lume.ufrgs.br/bitstream/handle/10183/153016/000938457.pdf?sequence=1.

[27] Leal SCFJ, de Souza AL, Bronkhorst EM (2015). Manual CAST: caries assessment and treatment.

[28] (MS) M da S do B. Examiner’s manual (2009). Projeto SB Brasil 2010. MS Brasília.

[29] R Core Team. R: A language and environment for statistical computing. R Foundation for Statistical Computing, Vienna; 2014. https://stat.ethz.ch/pipermail/r-help/2014-October/422975.html.

[30] Leal SC, Ribeiro APD, Frencken JE (2017). Caries assessment Spectrum and treatment (CAST): a novel epidemiological instrument. Caries Res.

[31] Maltz M, Alves L. S. G S, Moura M. S. Epidemiology of dental caries. In: Cariology: basic concepts, diagnosis and non-restorative treatment. São Paulo: Artes Médicas; 2016. p. 51–64. (ABENO).

[32] Cohen J. Statistical power analysis for the behavioral sciences. Hillsdale NJ: Erlbaum; 1988.

[33] FERGUSON CJ (2009). An effect size primer: a guide for clinicians and researchers. Prof Pathol Res Pract.

[34] Silva EMM, Sena D, Gutierres JCM, JHF G (2005). Quantitative analysis of subjectivity: an example of attribute agreement.

[35] Conboy JE (2012). Some typical univariate measures of magnitude of effect. Psychological Analysis.

[36] Pinto VG (2013). Public Oral Health.

