# Supplementary material for: Comparison of caries lesion detection methods in epidemiological surveys: CAST, ICDAS and DMF
Source: BMC Oral Health. 2018 Jul 6;18:122. doi: 10.1186/s12903-018-0583-6 (PMC6035475; doi:10.1186/s12903-018-0583-6)
Supplement: Supplementary file 1 — Appendices: Methods codes and intra-examiner and inter-examiner reproducibility. (DOCX 25 kb) [file 12903_2018_583_MOESM1_ESM.docx]

APPENDICES

**Table 5.** Intra-examiner and inter-examiner reproducibility measured by the Kendall concordance coefficient (Kendall’s W), using the DMF method in students, staff and their dependents at UNEB, Salvador, Bahia, Brazil, 2016.

| **DMF During calibration During the exams** |
| --- |
| (Kendall’s W) (Kendall’s W)  **n m sd n m sd**  INTER-EXAMINERS  Examiners 1, 2, 3 and 4 5 0.992 0.026 30 0.994 0.017  INTRA-EXAMINERS  Examiner 1 5 0.991 0.033 27 0.996 0.019  Examiner 2 5 0.991 0.032 27 0.997 0.014  Examiner 3 5 0.990 0.033 27 0.996 0.015  Examiner 4 5 0.994 0.023 27 0.997 0.018 |

**Table 6.** Intra-examiner and inter-examiner reproducibility measured by the Kendall concordance coefficient (Kendall's W), using the CAST method in students, staff and their dependents at UNEB, Salvador, Bahia, Brazil, 2016.

| **CAST During calibration During the exams**   \| (Kendall’s W) (Kendall’s W)  **n m sd n m sd**  INTER-EXAMINERS  Examiners 1, 2, 3 and 4 5 0.884 0.043 30 0.990 0.025  INTRA-EXAMINERS    Examiner 1 5 0.994 0.026 27 0.997 0.018  Examiner 2 5 0.996 0.021 27 0.996 0.015  Examiner 3 5 0.993 0.033 27 0.997 0.019  Examiner 4 5 0.994 0.028 27 0.995 0.027 \| \| --- \| |
| --- | --- |

**Table 7.** Intra-examiner and inter-examiner reproducibility measured by the Kendall concordance coefficient (Kendall's W), using the ICDAS method in students, staff and their dependents at UNEB, Salvador, Bahia, Brazil, 2016.

| **ICDAS During calibration During the exams** |
| --- |
| **(Kendall’s W) (Kendall’s W)**  **n m sd n m sd**  INTER-EXAMINERS  Examiners **1, 2, 3 and 4 5 0.804 0.043 30 0.983 0.027**  INTRA-EXAMINERS  Examiner 1 **5 0.989 0.039 27 0.991 0.032**  Examiner 2 **5 0.990 0.037 27 0.983 0.044**  Examiner 3 **5 0.989 0.040 27 0.982 0.042**  Examiner 4 **5 0.994 0.029 27 0.984 0.041** |

**Indices codes:**

1. **ICDAS codes:**
2. **ICDAS Caries Detection System:**
3. 0 - Sound
4. 1 - Opacity with air-drying white or brown
5. 2 - Opacity with air-drying white or brown
6. 3 - Enamel surface integrity loss
7. 4 - Underlying grey shadow
8. 5 - Dentine distinct cavity
9. 6 - Dentine extensive cavity
10. **ICDAS Restauration and selant coding system:**

0- Sound, surface not restored or sealed

1. Selant, partial
2. Selant, full
3. Tooth colored restoration
4. Amalgam restoration
5. Stainless-steel crown
6. Porcelain or gold or PFM (porcelain fused to metal crown)
7. Lost or broken restoration
8. Temporary restoration
9. Used for the following conditions

90- Implant for other non carious related reasons

91- Implant placed due to caries

92- Pontic placed for reasons other than caries

93- Pontic placed for caries reasons

96- Tooth surface cannot be examinated: surface excluded

97- Tooth missing because of caries

98- Tooth missing for reasons other than caries

99- Unerupted

1. Source: Ismail AI, Sohn W, Tellez M, Amaya A, Sen A, Hasson H, et al. The International Caries Detection and Assessment System (ICDAS): an integrated system for measuring dental caries. Community Dent Oral Epidemiol. 2007;35:170-8.
2. **CAST codes:**
3. Sound 0 - No visible evidence of a distinct carious lesion is present
4. Sealant 1 - Pits and/or fissures are at least partially covered with a sealant material
5. Restoration 2 - A cavity is restored with an (in)direct restorative material
6. Enamel 3 - Distinct visual change in enamel only; a clear caries-related discoloration is visible, with or without localised enamel breakdown
7. Dentine 4 - Internal caries-related discoloration in dentine; the discoloured dentine is visible through the enamel, which may or may not exhibit a visible localised breakdown
8. 5 - Distinct cavitation into the dentine; the pulp chamber is intact
9. Pulp 6 - Involvement of the pulp chamber; distinct cavitation reaching the pulp chamber, or only root fragments are present
10. Abscess/fistula 7 - A pus-containing swelling or a pus-releasing sinus tract related to a tooth with pulpal involvement
11. Lost 8 - The tooth has been removed because of dental caries
12. Other 9 - Does not match with any of the other descriptions
13. Source: Manual CAST: caries assessment and treatment. Holanda: Ipskamp Drukkers; 2015
14. **DMF Codes:**
15. 0 - Healthy - There is no evidence of caries. Initial stages of the disease are not taken into account
16. 1 - Carious groove, fissure or smooth surface has an evident cavity or softened tissue at the base or discoloration of the enamel or wall, or there is a temporary restoration
17. 2 - Restored but carious - There are one or more restorations, and simultaneously, one or more areas are decayed. There is no distinction between primary and secondary caries
18. 3 - Restored and without caries - There are one or more definitive restorations, and there are no primary or recurrent caries. A tooth with a crown placed due to decay is included in this category
19. 4 - Lost due to caries - A permanent or deciduous tooth has been extracted because of caries and not for other reasons
20. 5 - Loss for another reason - Absence is due to orthodontic, periodontal, traumatic or congenital reasons
21. 6 - Sealant - There is a fissure sealant, or the occlusal fissure has been enlarged to receive a composite
22. 7 - Support of the bridge or crown - Indicates a tooth that is part of a fixed prosthesis. This code is also used for crowns installed for reasons other than caries or teeth with aesthetic facets
23. 8 - Not erupted - The permanent tooth has not yet erupted. Does not include teeth lost due to congenital problems, trauma, etc.
24. Source: BRAZIL. Ministry of Health. Examiner's Manual. SB Brazil Project 2010. MS Brasilia; 2009.
